# Prevalence of *Candida* bloodstream isolates from patients in two hospitals in Vietnam

**Published:** 2019-04

**Authors:** Nguyen Duy Bac, Le Tran Anh, Le Bach Quang, Nguyen Khac Luc, Tran Thi Thanh Nga, Minoru Nagi, Miyazaki Yoshitsugu, Hoang Thi Thu Ha, Dang Duc Anh, Do Quyet, Do Ngoc Anh

**Affiliations:** 1 Department of Genetics and Cytogenetics, Institute for Military Medical Research, Military Medical University, Ha Noi, Vietnam; 2 Department of Medical Parasitology, Military Medical University, Ha Noi, Vietnam; 3 Department of Medical Bacteriology, Choray Hospital, Ho Chi Minh, Vietnam; 4 Department of Chemotherapy and Mycoses, National Institute of Infectious Diseases, Tokyo, Japan; 5 Department of Bacteriology, National Institute of Hygiene Epidemiology, Ha Noi, Vietnam; 6 Department of Tuberculosis and Lung Diseases, 103 Military Hospital, Military Medical University, Ha Noi, Vietnam

**Keywords:** Prevalence, *Candida*, Bloodstream, Vietnam

## Abstract

**Background and Objectives:**

Identification of yeasts provides helpful information for appropriate administration of anti-fungal treatments; however, few reports from the Vietnam have been published. This study has been performed to find the prevalence of *Candida* blood stream isolates from patients in two hospitals in Vietnam.

**Materials and Methods:**

*Candida* spp. were isolated from blood cultures in two hospitals, Vietnam between May 2013 and May 2015. Participating hospitals were 103 Military Hospital, Ha Noi city (550 beds) and Cho Ray Hospital, Ho Chi Minh city (1800 beds). All the bloodstream isolates were identified to species level by the germ tube test and polymerase chain reaction-restriction fragment length polymorphism (PCR-RFLP). In addition, unknown isolates were subjected to PCR sequencing.

**Results:**

A total of 93 *Candida* isolates were isolated from blood cultures during the study period. The results of this study showed that *C. tropicalis* (n = 47, 50.54%) was the most common agent, followed by *Candida albicans/dubliniensis* (n = 18, 19.35%), *C. parapsilosis* (n = 16, 17.20%), *C. glabrata* (n = 6, 6.45%), *C. mesorugosa* (n = 5, 5.38%) and *C. krusei* (n = 1, 1.08%), respectively.

**Conclusion:**

The frequency of the non-*albicans Candida* species in blood is increasing, especially *C. tropicalis*. Additional investigations should be made to clarify the epidemiological profile of invasive *Candida* bloodstream in Vietnam.

## INTRODUCTION

*Candida* species are major fungal pathogens of humans causing a diverse range of diseases (mucosal and invasive candidiasis, respectively) (1). Most notable among invasive candidiasis is candidemia, which is now the fourth most common bloodstream infection in the United States (1). Nosocomial bloodstream infections due to *Candida* species are associated with a mortality rate of 5% to 71% (2, 3). According to the findings of the Centers for Disease Control and Prevention (CDC), *Candida* infections are held responsible for 11% of all nosocomial infections (4). *Candida albicans* remains the leading cause of *Candida* bloodstream infections, however, the prevalence of non-*albicans Candida* infections has increased worldwide, particularly *C. tropicalis, C. glabrata, C. parapsilosis* and *C. krusei* (3). The species prevalence of *Candida* isolates varies between countries, regions, and institutions (5, 6). For example, the prevalence of *C. albicans* in Canada and Europe is higher than other regions (7). In the United States (US), there is a higher proportion of *C. glabrata* than other regions, while *C. tropicalis* was disproportionately prevalent in Latin America (7). Because of the different antifungal resistance profiles of *Candida* species and the widespread use of empirical antifungal therapy, treatment planning according to the likely effect is especially important in cases where antifungal susceptibility test can not be performed (5, 7). Besides, accurate epidemiology of invasive *Candida* infections is important to support institutional, national and regional guidelines for empiric treatment of suspected infection (8). Therefore, identifcation of the fungal strains is important for treatment and performance of infection control measures to prevent *Candida* infections (9).

Previously, there are many publications from Asian countries (6), but very limited data is available on the species distribution of *Candida* bloodstream in Vietnam. This study has been performed to find the prevalence of *Candida* bloodstream isolates from patients in two hospitals in Vietnam.

## MATERIALS AND METHODS

### Clinical isolates.

A total of 93 *Candida* bloodstream isolates obtained from patients were evaluated at the 103 Military Hospital (550 beds, Ha Noi city) and Cho Ray Hospital (1800 beds, Ho Chi Minh city) between May 2013 and May 2015. In order to isolate yeast colonies from blood cultures, the blood cultures were subcultured on Sabouraud dextrose agar (Merck, Germany) for 48 h at 30°C and suspended in sterile water at a concentration of 10^6^ CFU/ml (McFarland 0.5 corresponds to 1 - 5 × 10^6^ CFU/ml) (10). The yeasts were first identified according to morphological characteristics using germ tube test in sheep serum. After the initial morphological identification, yeast isolates were identified by PCR and sequencing.

### Germ tube test.

0.5 ml of sheep serum was put in a 1.5 ml sterile microcentrifuge tube. A sterile wooden applicator stick was used to pick a colony of yeast up and gently emulsified it in the serum. The tube was incubated at 37°C for 2 to 4 hours. After incubation, a drop of the suspension was placed on a clean microscopic slide with a coverslip. The wet mount was examined microscopically (at 40×) for production of germ tube (long tube-like projections extending out for yeast cells). *Candida albicans* ATCC 90028 was used for quality control.

### DNA isolation.

Pure cultures of all *Candida* strains were homogenized in 100 μl of sterile water (Corning, USA) and incubated with sorbitol buffer (1 M sorbitol, 100 mM EDTA, 14 mM β-mercaptoethanol) and 200UI lyticase (Sigma-Aldrich, USA) for 60 min at 30°C to disrupt the fungal cell wall. After that genomic DNA of each individual strain was isolated using QIAamp DNA Mini Kit (Cat.No51304, QIAGEN, Hilden, Germany), following manufacture recommendation. DNA concentration (ng/μl) was estimated using a NanoDrop^™^ 2000 Spectrophotometer at 260 nm (Thermo Fisher Scientific, USA).

### PCR amplification.

The PCR amplification of ITS1-5.8S-ITS2 rDNA regions was performed using ITS1 (5′-TCC GTA GGT GAA CCT GCG G-3′) and ITS4 (5′- TCC TCC GCT TAT TGA TAT GC-3′) (Integrated DNA Technologies, USA) (11). The components of PCR reaction were as follows: 5 μl of 10 × PCR buffer, 2.0 mM MgCl_2_, 5 μl of 2 mM dNTPs (0.2 mM of each), 0.3 μM each primer, 1.25 units of *Taq* polymerase (Thermo Scientific, USA), 5 μl of template DNA and molecular grade dH O up to 50 μl. Reaction mixtures were subjected to initial denaturation at 95°C for 5 min, followed by 35 cycles of denaturation at 94°C for 45 sec, primer annealing at 56°C for 45 sec and polymerization for 1 min at 72°C. Final extension step was performed at 72°C for 10 min. Sterile deionizer water used as negative control.

### RFLP.

RFLP was performed according to the method described by Mirhendi et al. to identify the most medically important *Candida* species (12). To perform RFLP assay, total volume of 16 μl, including 5 μl PCR product was added with 1 μl of *Msp*1 (10UI), 1 μl of 10 × Tango buffer (Thermo Fisher Scientific, USA) and 9 μl of distilled water. According to the manufacturer's instruction, the tubes were incubated at 37°C for 3 hours, to make sure full cutting of fragments. For analyzing the digestion products, 6 μl of each product in addition to 1 μl of loading dye buffer were electrophoresed on 2% agarose gel in 1× TBE buffer for about 2.0 h at 90V and visualized staining with 0.5 μg/ml of ethidium bromide on UV illumination (UVP, Canada). The size of each band was determined by a 100 bp plus ladder molecular weight marker (Thermo Fisher Scientific, USA).

### DNA sequencing.

Eight PCR products of our study were sent to First BASE Laboratories Sdn Bhd service (Kembangan 43300, Selangor, Malaysia) for purification and automatic sequencing in both directions of the D1/D2 rDNA regions. Sequences were read on ABI 3130 Genetic Analyzer software (seqscape software v2.1). The accuracy of data was confirmed by two-directional sequencing. Eight sequences were deposited in the GenBank under accession number MH891781 to MH891788.

### Statistical methods and sequence analyses.

Statistical Product and Service Solutions (SPSS) 20.0 software was used for data processing in our study. The sequences obtained were analyzed independently by comparing with related sequences available in the GenBank database, using BLAST guidelines (http://blast.ncbi.nlm.nih.gov/Blast.cgi).

## RESULTS

A total of 93 *Candida* bloodstream isolates (85 isolates from Cho Ray Hospital and 8 isolates from 103 Military Hospital) obtained from patients were subjected to germ tube test. The test used to differentiate between *C. albicans* and non-*albicans Candida*. The results are shown in Table 1.

**Table 1.** Frequency of *Candida* identified by germ tube test

**Species**	**Number**	**Frequencies**
*C. albicans/C. dubliniensis*	17	18.28
non-*albicans Candida*	76	81.72
Total	93	100

**Fig. 1.**
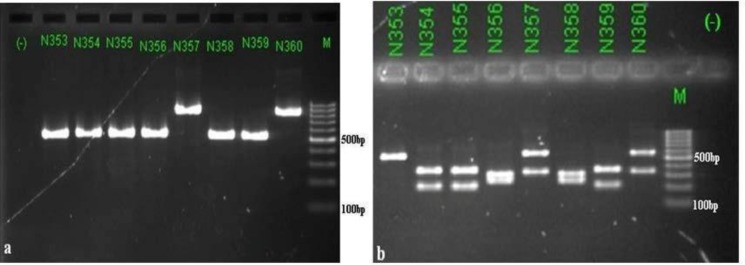
Patterns of PCR products of *Candida* isolates before (Fig. 1a) and after digestion with the restriction enzyme *MspI* (Fig. 1b). land (−): negative control; land N353: *C. parapsilosis*; land N354 (MH891783), N355, N359: *C. tropicalis*; land N356, N358: *C. albicans*; land N357 (MH891784), N360: *C. glabrata*; land M: 100 bp DNA ladder.

By PCR-RFLP method, *C. tropicalis* was the pre-dominant species (n = 47, 50.54%); followed by *C. albicans* (n = 18, 19.35%), *C. parapsilosis* (n = 16, 17.20%), *C. glabrata* (n = 6, 6.45%) and *C. krusei* (Fig. 1) (n=1, 1.08%). Five fungal strains were classified as uncommon yeast species (Table 2). After sequencing and comparing with related sequences available in the GenBank database, these 5 isolates were identified as *C. mesorugosa.*

**Table 2.** Frequency of *Candida* identified by PCR-RFLP

**Yeastspp.**	**Number**	**Frequencies**
*C. tropicalis*	47	50.54
*C. albicans/C. dubliniensis*	18	19.35
*C. parapsilosis*	16	17.20
*C. glabrata*	6	6.45
*C. krusei*	1	1.08
*Candida* sp.	5	5.38
Total	93	100

We deposited eight sequences of the D1/D2 rDNA regions of *Candida*, including one of *C. albicans* strain and seven non-*albicans Candida* strains, in the NCBI database (GenBank, USA) under accession number MH891781 to MH891788.

## DISCUSSION

Rapid identification of *Candida* species in clinical laboratory is becoming increasingly important since the incidence of Candidiasis are growing up and different *Candida* species have been resistant to antifungal drugs (9). *Candida* spp. are the fourth most common cause of all nosocomial bloodstream infections and the third most common cause of nosocomial bloodstream infections in the intensive care unit (ICU) setting in U.S. hospitals (13). High mortality associated with candidemia can be reduced by prompt, appropriate antifungal therapy. A delay in the initiation of fluconazole therapy in hospitalized patients with candidemia is significantly impacted mortality (14).

According to the results, 18 strains of yeasts were identified as *C. albicans/C. dubliniensis* and 75 strains were non-*albicans Candida* with germ tube test and PCR-RFLP. Although approximately 5% of *C. albicans* isolates failed to produce a germ tube when incubating at 37°C (15). However, germ tube testing is a rapid and simple test to achieve a presumptive identification of *C. albicans* in routine laboratory (16). During a period between 2–4 hours, *C. albicans* will form germ tube at 37°C while other species not produce germ tube. *C. dubliniensis* also produces germ tubes in the germ tube test (17). In fact, *C. albicans* and *C. dubliniensis* are phenotypically similar (18). Morphological characteristics and PCR-RFLP with *Msp*I enzyme are often quite difficult to discriminate between these two species in clinical samples (12, 18). An additional tests, therefore are required for differentiating from *C. albicans* (17). In this study, we do not discriminate *C. albicans* and *C. dubliniensis*. A reason for that is in most analyses of systemic infection, *C. albicans* is found in > 50% of cases, while if it is identified at all, *C. dubliniensis* has only been found in at most 2–3% of cases, and is rarely resistant to azole (13).

The results of this study showed that the frequency of non-*albicans Candida* accounted to 80.65% of the total *Candida* isolates and were more common than *C. albicans/C. dubliniensis* (19.35%). In one of the similar studies performed in Malaysia, Mohamed et al. reported that out of yeast isolates from blood, 20.5% were *C. albicans* and 79.5% were non-*albicans Candida* (19). In a multi-centre study conducted by Tan et al. in Asia including China, Hong Kong, India, Singapore, Taiwan and Thailand, non-*albicans Candida* were seen at higher rate than *C. albicans* (6). The proportion of *Candida* species vary considerably among the patients with different risk factors as well as in different geographic areas (20). The incidence of non-*albicans Candida* worldwide is generally observed to be increasing (19). Our finding is also consistent with previous analyses in the other countries in Asia such as Japan (7), and Kuwait (21).

*C. tropicalis* was the species most commonly isolated from *Candida* bloodstream infections (n = 47; 50.54%). According to Tan et al. (2015), *C. tropicalis* candidaemia appears to be more common in tropical countries as India, Singapore and Thailand (6). The epidemiological data from India showed that *C. tropicalis* was the most dominant agent of candidaemia cases due to non-*albicans Candida* (22). *C. tropicalis* exhibits lower virulence than *C. albicans* because of its lower capability of adherence to epithelial cells but exhibit a great degree of variation in their antifungal susceptibility profile especially strains isolated from blood cultures (23). Therefore, proactive monitoring of fluconazole susceptibility is necessary in regions where *C. tropicalis* predominates.

The second most frequent of non-*albicans Candida* was *C. parapsilosis* (16; 17.20%). When we compared our results with other studies in Malaysia and Korea, we found that prevalence of *C. parapsilosis* was higher (19, 24). According to Wu Y et al. (2015), the rate of *C. parapsilosis* has dramatically increased and become the second most commonly isolated *Candida* species from blood cultures (25). *C. parapsilosis* is the most common one with decreased susceptibility to echinocandins and there is a correlation between increased caspofungin usage and increased *C. parapsilosis* candidemia (26) but in Vietnam those drugs are not common.

*C. glabrata* was the fourth most frequently isolated *Candida* species (6.45%). Although it accounted for a small proportion but *C. glabrata*, along with *C. krusei*, is the most frequent species with reduced susceptibility to one or several azoles (19, 21). In our study, only one isolate was identified as *C. krusei*. The results of our study were similar to those of a previous research in Kuwait (21) and Malaysia (19). In Japan, *C. glabrata* was the third most common species (7), where as *C. glabrata* was the most frequently isolated non-*albicans Candida* species in the USA and UK (13, 27). The reasons for such differences in the rate of *C. glabrata* might be explained the insvestigation of different regions and the number of *Candida* bloodstream isolates in these studies.

In the current study, five strains of unknown species were identified and confirmed by sequencing assay. All of them were *C. mesorugosa*, a species of *C. rugosa* complex. *C. rugosa* complex has been described as an emerging human fungal pathogen, which has been most frequently recorded in Latin America (2.7% of all *Candida* spp.) versus other regions of the world (0.1–0.4%) (28, 29, 30). *C. mesorugosa* has been isolated from various human sources including blood, rectal swabs, pericatheter swabs and blood sample (29, 31). The prevalence of un-common *Candida* spp., such as *C. rugosa* complex, vary by geographic region, patient population, and antifungal practices (20, 30, 32). *C. rugosa* isolates from blood cultures had been reported in Asian as Malaysia (19) and Vietnam (33). Nevertheless, there are no reports of *C. mesorugosa* from Vietnam exist. This is the first report of blood infections caused by *C. mesorugosa* in Vietnammese.

## CONCLUSION

The frequency of the non-*albicans Candida* species isolated from blood is gradually increasing and rapid and precise identification of *Candida* species will help clinicians make an appropriate therapeutic. Additional investigations should be made to clarify the epidemiological profile of invasive *Candida* bloodstream in Vietnam.
